# Detection of Aspens Using High Resolution Aerial Laser Scanning Data and Digital Aerial Images

**DOI:** 10.3390/s8085037

**Published:** 2008-08-25

**Authors:** Raita Säynäjoki, Petteri Packalén, Matti Maltamo, Mikko Vehmas, Kalle Eerikäinen

**Affiliations:** 1 University of Joensuu, Faculty of Forest Sciences, P.O.Box 111, FI-80101 Joensuu, Finland; E-mails: raita.saynajoki@joensuu.fi, petteri.packalen@joensuu.fi, matti.maltamo@joensuu.fi, mikko.vehmas@joensuu.fi; 2 Finnish Forest Research Institute, Joensuu Research Unit, P.O Box 68, FI-80101 Joensuu, Finland; E-mail: kalle.eerikainen@metla.fi

**Keywords:** Airborne laser scanning, digital aerial images, aspen, individual tree detection, tree species classification

## Abstract

The aim was to use high resolution Aerial Laser Scanning (ALS) data and aerial images to detect European aspen (*Populus tremula* L.) from among other deciduous trees. The field data consisted of 14 sample plots of 30 m × 30 m size located in the Koli National Park in the North Karelia, Eastern Finland. A Canopy Height Model (CHM) was interpolated from the ALS data with a pulse density of 3.86/m^2^, low-pass filtered using Height-Based Filtering (HBF) and binarized to create the mask needed to separate the ground pixels from the canopy pixels within individual areas. Watershed segmentation was applied to the low-pass filtered CHM in order to create preliminary canopy segments, from which the non-canopy elements were extracted to obtain the final canopy segmentation, i.e. the ground mask was analysed against the canopy mask. A manual classification of aerial images was employed to separate the canopy segments of deciduous trees from those of coniferous trees. Finally, linear discriminant analysis was applied to the correctly classified canopy segments of deciduous trees to classify them into segments belonging to aspen and those belonging to other deciduous trees. The independent variables used in the classification were obtained from the first pulse ALS point data. The accuracy of discrimination between aspen and other deciduous trees was 78.6%. The independent variables in the classification function were the proportion of vegetation hits, the standard deviation of in pulse heights, accumulated intensity at the 90^th^ percentile and the proportion of laser points reflected at the 60^th^ height percentile. The accuracy of classification corresponded to the validation results of earlier ALS-based studies on the classification of individual deciduous trees to tree species.

## 1. Introduction

High resolution remote sensing data enable the interpretation of forests at the tree level. By using airborne laser scanning (ALS) data with a pulse density of several pulses per square metre and aerial images with a resolution of less than 50 centimetres, it becomes possible to detect individual trees and accurately classify them by species [1-8].

ALS data have been used in remote sensing since the 1980's [9]. In order to apply the technique to the delineation of individual trees, however, it is necessary to construct a canopy height model (CHM) capable of distinguishing the tree crowns from each other [4]. In general, the methods used in laser scanning-based single tree detection are only comparable to those applied in high or very high-resolution aerial imagery-based surveys [1, 6]. For individual tree detection based on searches for local maxima, a low-pass filtered CHM is needed, due to large number of false local maxima in an unfiltered model.

Although ALS data can be successfully used to detect individual trees and measure the layer of dominant trees, the results regarding its applicability to suppressed tree layers have been less promising [5, 7]. The tree crowns of suppressed trees are usually partly or completely covered by the crowns of larger trees, and therefore their tops are hidden from the ALS point cloud. ALS-based detection of individual trees has been studied, for instance, by Hyyppä and Inkinen [10], Persson *et al.* [3], Pitkänen *et al.* [6], Koukoulas and Blackburm [11], Solberg *et al.* [7] and Koch *et al.* [12].

Persson *et al.* [3] used Gaussian filtering for image smoothing and a region growing method to detect individual trees from a canopy height model generated from ALS data for an area that consisted mainly of middle-aged or old coniferous forest. The detection rate was 71% for all trees and 90% for trees with a DBH over 20 cm.

In their comparison of smoothing methods, Pitkänen *et al.* [6] defined sample plots in mature forests with stand volumes from 127 to 533 m^3^/ha. Many of the stands had a multilayered canopy structure. The methods used were Gaussian filtering, height-based filtering, elimination of maxima and Laplacian filtering. The identification rates for all trees varied from 36.7% (Gaussian filtering) to 41.5% (elimination of maxima), and those for dominant trees from 61.2% (height-based filtering) to 68.7% (elimination of maxima).

Koukoulas and Blackburn [11] detected 80% of trees in a semi-natural forest using a CHM derived from ALS data and extracting treetops by a contouring method. Solberg *et al.* [7] developed a region growing algorithm for delineating the tree segments and succeeded in detecting 93% of the dominant trees altogether and 19% of the suppressed trees.

A classification of tree species with high-resolution ALS data can be based on: 1) the features of crown shape and characteristics of pulses reflected from the crown [5]; 2) proportions of canopy areas of dominant tree species, using linear discriminant analysis [13]; 3) directed graphs describing instances of laser points of single tree segments and resulting point groups [14]; 4) segments delineated with a digital surface model generated from leaf-on ALS data [15]; 5) the use of leaf on-off data [16], and 6) ALS intensity values [17].

It is also possible to combine ALS data with aerial photographs for automatic detection of both trees and their species. Packalén and Maltamo [18], for instance, estimated accurate species-specific stand variables at the plot level. Persson *et al.* [19] divided tree species into three classes at the tree level by combining near-infrared images with tree crown delineation with ALS data. Korpela [8] detected the species of individual trees by visual interpretation of aerial photographs, combining this information with single tree ALS data.

The European aspen (*Populus tremula* L.), referred to below as 'aspen', is commonly found throughout Finland, but pure aspen or aspen-dominated forests are rare [20]. Its growth is most rapid in fertile stands, where trees can reach a height of 30 metres and a diameter at breast height of 90 cm. According to Kouki *et al.* [21], large aspen are hosts for hundreds of herbivorous and saproxylic invertebrates, polypore fungi and epiphytic lichens, many of which are threatened species and about 150 are strictly specialised to aspen. There are also vertebrates such as woodpeckers [22] and the flying squirrel [23] that are partly dependent on aspen. This means that large aspens are extremely valuable for maintaining the biodiversity of boreal forests. In the past, however, forest management practices discriminated against large aspens and aspen-associated species, so that aspen frequencies are low even in recently established conservation areas [21].

The aim of this work was to apply ALS data and aerial photographs to the discrimination of aspens from other deciduous trees. In particular, we concentrated on identifying large aspens (diameter at breast height >25 cm). Following the delineation of individual trees using a filtered CHM based on tree heights derived from ALS data, the deciduous trees were separated from the conifers by visual interpretation of digital aerial photographs. Finally, the deciduous tree segments were classified into aspens and other species by linear discriminant analysis using predictor variables obtained solely from the ALS data.

## 2. Materials and preprocessing

### 2.1 Field data

The field data were obtained from 14 sample plots of 30 m × 30 m size located in the Koli National Park in Eastern Finland. The locations of the sample plots were selected to maximise the variation in the structure, age and number of tree species of the forest stands containing aspen. Other tree species represented in the data were Scots pine (*Pinus sylvestris* L.), Norway spruce (*Picea abies* (L.) H. Karst.), silver birch (*Betula pendula* Roth), downy birch (*Betula pubescens* Ehrh.) and alder (*Alnus spp.*). The plots included the following forest types: 1) nutrient-rich forests (*Oxalis-Maianthemum*-type, OMaT) 2) upland forests with grass-herb vegetation (*Oxalis-Myrtillus*-type, OMT) and 3) fresh mineral soil forests (*Myrtillus*-type, MT) (Cajander 1926). Most of the plots were located on mature or multilayered forest stands, but some were in middle-aged stands.

The tree-level field measurements of diameter at breast height (DBH, mm) of trees larger than 5 cm, diameter at six metres (D_6_, mm), species, height (H, dm) and crown height (H_c_, dm) were carried out in September 2005, in connection with which the trees were also classified as living or dead and belonging to the dominant or suppressed tree population. In addition, the *x* and *y* coordinates of all trees were recorded in the field. Stem volumes were calculated using the volume functions of Laasasenaho [24], which included DBH, H and D_6_ as predictors. Since these functions are intended for use with Scots pine, Norway spruce and birches, the volume estimates for aspens with a diameter at breast height smaller than 20 cm were calculated using the function for birches, whereas those for aspens with a diameter at breast height of 20 cm or larger were obtained using the function for pine, as suggested by Kinnunen *et al.* [25]. The stem volumes of the other deciduous trees were calculated with the function for birch.

The descriptive statistics for characterization purposes were obtained at the stand level (Table 1). The lowest and highest values obtained for the percentage of aspen in the total stand volume were 13% and 78%, respectively, whereas the percentages of aspen in the total number of trees varied from 3% to 46%.

Differentially corrected Global Positioning System (GPS) measurements were used to determine the position of the four corners of each of the 14 plots. The accuracy of positioning in the x/y direction was about one metre. Tree locations within a plot were measured using one corner as the origin. Finally, all the trees on each plot were projected into the coordinate system of the ALS data by means of the affine transformation, using the measured corner positions as reference points.

### 2.2 Remote sensing data

The ALS data were collected using an Optech ALTM 3100 laser scanning system in the middle of July 2005. A total of nine flight lines were run with a flight altitude of 900 metres, a flight speed of 75 m/s and a scan angle of 11 degrees. Given a pulse repetition rate of 100 kHz, a nominal pulse density of 3.86 pulses/m^2^ was achieved. The x, y and z-coordinates, the number of the flight line, the intensity and pulse type (only echo, first of many echoes, intermediate echo, and last of many echoes) were recorded for every reflected pulse. The data were supplied in the EUREF-FIN coordinate system.

In the digital terrain model (DTM), which was interpolated from the last pulse data using the method described by Axelsson [26], every pixel was assigned a height relative to the geoid. Using the point cloud of the DTM, the CHM was then interpolated according to the maxima within a radius of 0.5 metres. The height values of the pixels in the CHM were expressed relative to ground level.

Aerial images were captured with a Vexcel UltraCamD digital frame camera in September 2005. The area was covered by the two flight lines of length 10 kilometres, and images were taken at an altitude of 3,000 metres above ground level with a sidelap of 67% and an endlap of 80%. As a part of the standard data processing, chains of the Vexcel UltraCamD multispectral bands (red, green, blue, NIR) were fused with the higher-resolution panchromatic band by means of the pan-sharpening procedure. Only the pan-sharpened NIR, red and green bands are used in this paper, however. The pan-sharpened images were finally orthorectified by reference to the ALS-based DTM and resampled to a resolution of 25 cm.

## 3. Methods

### 3.1 Low-pass filtering

The purpose of the low-pass filtering of the CHM was to smooth out high-level frequencies in order to reduce the number of local maxima and increase the proportion of true local maxima, i.e. actual tree tops. The main goal was to detect large aspens as accurately as possible. A smoothing method proposed by Pitkänen et al. [6] was used. It relies on kernels which are based on Gaussian distribution:

(1) $$G\left(x,y\right)=\frac{1}{2\pi \sigma^{2}}e^{-\frac{x^{2}+y^{2}}{2\sigma^{2}}},$$

where *x* and *y* are the distances from the kernel centre and σ is the standard deviation of the distribution. The size of the smoothing window and the intensity of smoothing increase stepwise as a function of the heights of the CHM. The size of the window is smallest and the smoothing mildest in the lowest class, and correspondingly, the smoothing is always most intense at the greatest heights. The parameters required in the height-based filtering include a sigma (σ) and a class height. Here a fixed class height of 6 metres was used, and the sigma (σ) parameter was given for the lowest (0-6m) and highest height classes, allowing its value to change stepwise at intervals of 6 metres between these extremes. Individual sigma values were obtained visually for every sample plot. Sigma values and the number of height classes are presented in Table 2.

### 3.2 The search for local maxima and binarization

Local height maxima in the low-pass filtered CHM were first searched for by a method in which all the pixels were initially marked as possible maxima [6], after which they were examined iteratively and any pixel having a neighbour (in an eight-connected neighbourhood) with a higher value was labelled as a non-maximum. Local maxima were then identified in the highest sections of the CHM and also in the lowest sections (ground), and finally the maxima in the highest sections were taken as representing tree tops, whereas those in the lowest sections were masked out by the binarization.

In the binarization, all the pixels were classified as belonging either to the tree canopy or to the background area by defining a threshold value, chosen separately for each plot. As the binarization was performed on the smoothed CHM, fairly high threshold values were needed in order to eliminate the undergrowth from the local maxima in the background area.

### 3.3 Segmentation

Watershed segmentation was performed on the smoothed CHM. Watershed algorithms are counted among the hybrid techniques which combine boundary and region-based methods [27]. In watersheds, an image is visualized in three dimensions: x and y-coordinates and grey levels [28]. The image is regarded as a topographic surface, where the darkest grey values represent low points and the brightest ones the top points of the surface. Starting from the minimum values of the image, the surface is filled with water. Basins surrounding two of the minima may merge at some points. To avoid this, a dam consisting of single pixels is built on the edge of the basins. Finally, all the basins are bounded by dams, which constitute the boundaries of the segments [28, 29]. Watershed algorithms produce closed boundaries even if the transitions between areas are not equally strong [27].

Since the algorithm used here processed the negative of the CHM, the segmentation was started from the local minima, which were actually the local maxima of the CHM, i.e. the assumed top points of the canopies. Pixels belonging to the local minima were labelled with a new segment number, whereas pixels not belonging to the minima were linked to their neighbouring pixels with the smallest pixel value. Each pixel was linked to one minimum by following the path already formed. After this the flooding algorithm was implemented.

Finally, the binarization image and the segmentation were combined, the pixels labelled as the background in the thresholding being set as background in the segmentation image as well. Thus the canopy segments did not include any pixels with a height value smaller than the threshold value, and local maxima lying outside the canopy were not taken into account. No parameters need to be chosen in the watershed segmentation itself, but because the operation is performed on a filtered CHM, the intensity of the low-pass filtering defines the quality of segmentation.

### 3.4 Linking local maxima with field trees

The height value of every local maximum was compared with the nearby measured tree heights. If height values were almost equal, and the xy-distance between the maximum and the measured tree coordinates was not too much, the measured tree was linked to the local maximum and its canopy segment. More variation in heights (z) and distances (xy) was allowed among the large trees. In some cases the measured heights were allowed to be about four metres greater than the local maximum z value in the CHM, but measured heights that were less than the values of the local maxima were restricted to an accuracy of two metres. The mean distance was 1.21 metres in the z direction and 1.38 metres in the xy direction between field coordinates and local maxima.

### 3.5 Classification of coniferous and deciduous trees

The trees were classified into coniferous and deciduous visually using aerial images. Tree segments and the corresponding local maxima were overlaid with aerial images on the screen. Due to radial displacement the segments and the canopies in aerial images did not match perfectly, but as radial displacement is strongest near the edges of the image, only images with a plot located not very far from the nadir were used. For the most of the plots two or three images were used for classification. This work was done by a person who was not familiar with the data but who had worked with aerial images before. All the segments were gone through and labelled as coniferous or deciduous trees.

### 3.6 Classification of aspen and other deciduous trees

A classification of aspen and other deciduous trees was based on ALS data only. The first step was to convert ALS data to above-ground scale by subtracting the DTM from the orthometric heights. The ground hits were excluded by assuming that any point with a canopy height less than 0.5 metres is a ground hit and that the remaining points are canopy hits. After that the ALS points were extracted from the tree segments and height distribution was created for each tree. Only the first pulse data was used in the separation of aspens from the other deciduous trees.

The variables calculated for each tree segment were 5, 10, 20, …, 90, 95 percentiles of the laser height (m), the proportion of laser points reflected at each percentile relative to all first pulses, the proportion of vegetation hits, average height, standard deviation, average intensity, standard deviation of intensity, intensity values at the 10^th^, 20^th^, …, 90^th^ intensity percentiles, and the ratio between crown area and height. More predictors were created by using ratios between percentile heights and proportions. Since the mean height of the aspens (24.1 m) was greater than that of the other deciduous trees (20.5 m), the height percentiles were not used directly in the classification, in order to avoid excessively optimistic results.

A linear discriminant analysis was performed to distinguish the aspens from the other deciduous trees. To test the overall relationship between the groups to be discriminated and the predictors, cross-product matrices were formed [30]. A cross-product matrix associated with differences between groups and within groups can be determined as follows:

(2) $${\text{S}}_{\text{total}}={\text{S}}_{\text{bg}}+{\text{S}}_{\text{wg}}$$

where S_bg_ refers to systematic variation and S_wg_ to unexplained variation [31]. An *F* ratio is calculated to test whether the groups can be distinguished on the basis of the predictors (the null hypothesis is rejected) [30, 31]. If the Lambda for the test of the overall relationship does not reject the null hypothesis, it is not relevant to investigate the canonical discriminant functions any further, But if the *F* ratio exceeds the critical *F* and the p-value shows significance, further investigations can be made [31]. In the two-group case, i.e. aspens versus other deciduous trees, the canonical discriminant function is as follows [31]:

(3) $$Z=a_{1}X_{1}+a_{2}X_{2}+\ldots +a_{p}X_{p}\;,$$

where *a*_1_−*a_p_* and *X*_1_−*X_p_* are coefficients and predictor variables, respectively.

The function *Z* has a characteristic value λ which is obtained by comparing the within and between-sums-of-squares for the two groups. This leads to Wilks' lambda (Λ) and to the *F*-relation, which is to be maximized when choosing values for the parameters *a*_1_-*a_p_* [31]. The ALS-based predictors used in the discriminant analysis were chosen by a stepwise method based on Wilks' lambda [32]. The criterion for the choice of a predictor was the probability of the significances of *F*: 0.01 for the enter value and 0.05 for the remove value.

## 4. Results

The detection of individual trees was based on the height value of a local maximum and the heights of the nearby trees as measured in the field. 27% of the trees were detected overall, while the highest and lowest proportions at the plot level were 47% and 13%. The total proportion of true maxima was 79%, with values varying from 56% to 96%. 64% of the trees with a diameter at breast height of 15 cm or more were correctly detected with the HBF data. At the plot level a maximum of 79% of the trees were detected, whereas the lowest detection rate was 44%. When only trees with a DBH of 25 cm or more were considered, 87% were detected correctly, the detection rates for individual plots varying from 75% to 100%.

The detection rate for individual aspens was 57%, and varied from 27% to 100% between plots. When considering aspens with a diameter at breast height of 15 cm or more, 81% were detected, varying from 43% to 100% at the plot level. The overall proportion for aspens with a DBH of 25 cm or more was 96%. The total number of these large aspen was 90, and 86 of them were detected. There was at least one large aspen on every plot.

Manual classification of the trees into deciduous and coniferous using the digital aerial images, led to an overall success rate of 74% (Table 3), ranging at the plot level from only 55 % on plot 6 to 96% on plot 7.

The data used to distinguish the aspens from the other deciduous trees consisted of correctly detected and correctly classified deciduous trees, altogether 140 aspens and 56 other deciduous trees. The average values of the tree variables DBH, H, D6 and H_c_ were on average greater for the aspens than for the other deciduous trees, and their standard deviations were also correspondingly greater, with the exception of H. Also, the mean value of H_c_/H obtained for the aspens was higher than for the other deciduous trees, which means that the aspens had relatively shorter crowns, while the standard deviation of the H_c_/H values was lower for the aspens.

The maximum absolute value of the Pearson correlation in the estimated discriminant function was set at 0.60. Altogether 78.6% of the trees were correctly classified with the function:

(4) Z=−21.093−0.825×f_hstd+0.039×f_i90−0.105×f_veg+0.476×f_p60

where *f_veg* is the proportion of vegetation hits, *f_hstd* is the standard deviation in the heights of the reflected first pulses, *f_i*90 is the intensity value at the 90^th^ percentile, and *f_p60* is the proportion of laser points reflected at the 60^th^ height percentile. The proportions of successful classifications of aspens and other deciduous trees using Eqn (3) were 82.9% and 67.9%, respectively.

**Figure 1.**
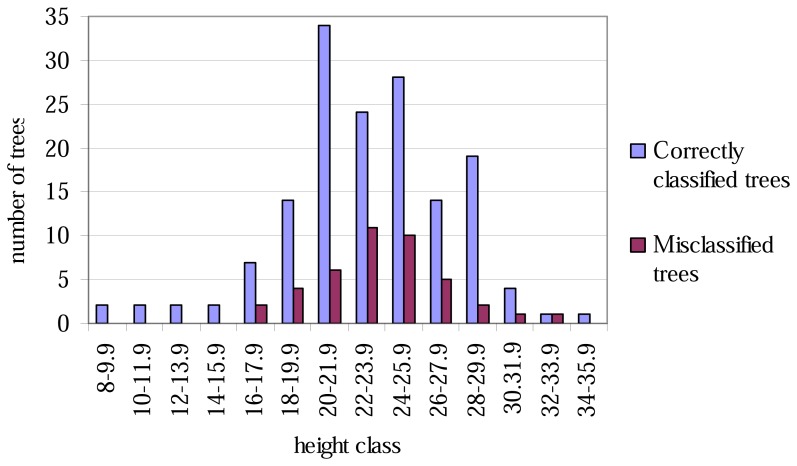
Correctly classified and misclassified trees, by 2-metre height classes.

**Figure 2.**
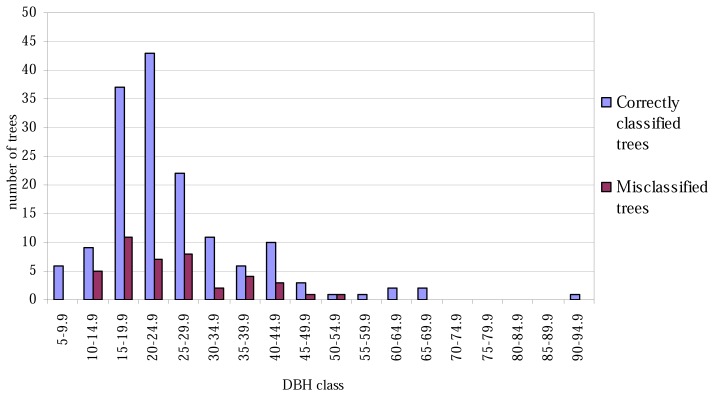
Correctly classified and misclassified trees, by 5-cm DBH classes.

The lowest classification accuracy at the plot level was only 60% (Table 4). No trees of height less than 16 metres were misclassified (Figure 1), and correspondingly, none of the tree with the smallest or largest DBH values were misclassified (Figure 2).

## 5. Discussion

The aim here was to discriminate aspens from other deciduous trees by a method that involved the delineation of individual trees based on ALS data, separation of the deciduous from the coniferous trees by reference to digital aerial photographs, and finally separation of the aspens from the other deciduous trees using ALS-based data variables. The first phase consisted of filtering of the CHM, searching for maxima in this model, binarization of the smoothed CHM and segmentation of the detected tree canopies. The trees were classified into coniferous and deciduous visually from digital images, whereas the classification into aspens and other deciduous trees was performed by linear discriminant analysis using the first pulse ALS data variables as predictors.

The test area was challenging for individual tree detection because of the multi-layered canopy structure and the size of the large aspens. The classification into aspens and other deciduous trees succeeded reasonably well, however, as 82.9% of the aspens were correctly identified. Only 57% of the aspens were found in the tree delineation phase, but the proportion was still 96% in the case of large aspens (DBH> 25 cm).

The choice of parameters for the low-pass filtering was mainly based on their ability to improve the accuracy of aspen detection. The parameters were chosen in order to obtain a smoothing for large aspens so as to have only one local maximum. If there was a high variation in the size of aspens on the plot, the largest ones were favoured. Since, large aspens often had several maxima in light smoothing, quite intense smoothing was needed for many plots. The natural result was that the smoothing became less efficient for other trees, i.e. smaller ones, since less local maxima are obtained with intensive smoothing and less trees are detected [10]. After low-pass filtering, a search for local maxima was performed and the height values of the detected trees were compared with the heights of those measured in the field. This comparison indicated roughly whether the maxima were true canopy tops or not.

**Figure 3.**
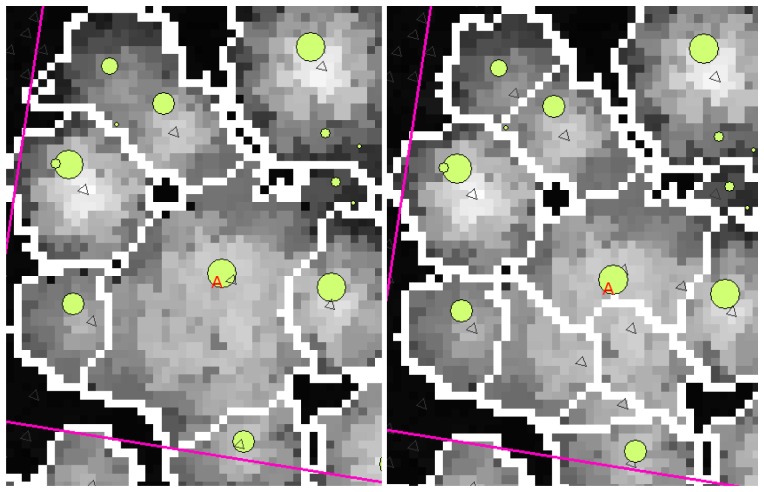
An example of the effects of smoothing intensity. There is an unsmoothed CHM in the background. Trees measured in the field are marked with circles of a size related to their DBH. The boundaries of the canopies are the result of the segmentation. A large aspen has been marked with an A and local maxima are marked with triangles. The smoothing parameter σ had the value 0.40 in the lowest height class and 2.20 in the highest in the left-hand image, the corresponding σ-values in the right-hand image being 0.40 and 1.20.

A clear effect of the intensive filtering on the resulting segmentations was the loss of true local maxima. Figure 3 is a typical example of the effects of intensity on smoothing. In the left-hand image the smoothing was so intense that the large aspen (marked 'A') was given only one local maximum, and therefore the segment became large enough. In the right-hand image, however, the smoothing is lighter and the canopy of the large aspen has four local maxima and four separate canopy segments. On the other hand, at least one local maximum is lost from the upper left-hand corner of the image and another from north-east of the aspen marked in the left-hand image. Some trees have been lost because their canopies have been joined to the crowns of neighbouring trees, resulting in one larger segment. This was the situation in the case of many of the plots. In order to obtain only one local maximum from a canopy, an increased intensity of smoothing is needed for the large aspens, which will simultaneously reduce the accuracy of segmentation for the other trees.

In the detection of individual trees, the heights of the detected trees as measured in the field were restricted to being no more than four metres greater or two metres less than their laser height counterparts. When the heights measured in the field were less than the laser heights, it was likely that a mistake had been made in measuring the height or that the local maximum had reflected from a neighbouring tree. It did indeed prove difficult to measure the heights of the tallest aspens with circular canopies in the field, and sometimes it was impossible to see the top of the tree because of the extent of the crown, which definitely detracted from the accuracy of the field measurements and later caused difficulties in the detection of individual trees.

It is a typical situation in laser scanning, that the laser beam does not reflect from the highest tree top, and this causes underestimation in laser heights [3, 5, 33, 34]. There is also a possibility of failures in laser height measurements caused by inaccuracies in the DTM. If there are gaps in the last pulse data, the interpolated DTM will have pixels with no actual ground reflections. The discrepancies between the real ground value and the interpolated value in 'no data'rsquo areas can be substantial with high gradients, as found on our plots. Hyyppä and Inkinen [10], using laser scanning data to measure tree heights under Finnish conditions, achieved an accuracy better than 1.0 m, whereas Maltamo et al. [5] managed an accuracy of 0.97 m. Altogether 46% of the tree heights in the latter case were underestimated by more than 1 metre, 37.4% by less than 1 metre, and 16.6% were overestimated due to errors in the field measurements [5]. In the present material the heights were underestimated on average by 1.2 metres.

The maximum differences between the tree base positions measured in the field and the laser-measured tree top positions were almost five metres (average 1.4 m). It is obvious that the difference can be a matter of several metres, especially among tall trees. The top of a tall tree can be slightly to one side of the base, and many of the present plots had a varying topography, so that some of the trees would have been leaning. A greater difference was therefore allowed among the tallest trees than among the smaller ones. The present differences between the trees measured in the field and the laser maxima were of the same order as the deviations reported by Hyyppä and Inkinen [10], less than 1.5 m.

Persson *et al.* [3] detected 71% of all trees by means of laser scanner data when their field data were derived from middle-aged and old spruce and pine-dominated forest stands, while Pitkänen *et al.* [6] found about 40% of all trees in “mainly mature, heavily stocked forest stands, many of which had multi-layered canopy structure”. Pitkänen *et al.* [6] also compared several smoothing methods, including HBF and Gaussian, which gave detection rates of 36.7% and 37.0%. About the same accuracy level was achieved by Heurich *et al.* [35], applying an algorithm developed for Swedish forests to an area in south-eastern Germany, as they detected 44% of the trees, with 5.4% false identifications. The accuracy of their method was highest in pure spruce stands and lowest in dense beech and spruce stands. Koukoulas and Blackburn [11] detected 80% of trees in semi-natural forest using ALS data, delineating the canopies by contouring and manipulating the resulting polygons. In the present study, 27% of the 1540 trees were detected and the accuracy was related to the stand density. The low detection rates can partly be explained by the intensive low-pass filtering, and another explanation could be that small trees are almost impossible to detect when the are located under taller, mature trees [5]. The area concerned was situated in a national park, and most of the plots were in a natural or semi-natural state with a multilayered canopy structure. The larger trees were detected with greater success, and the proportion of successfully detected aspens (57%) was considerably higher than the overall detection rate. This is partly explained by the preponderance of small trees, as the proportion of trees with a DBH of 15 cm or more was higher among the aspens (64%) than for all trees (38%). The low proportion of trees detected on some plots was due to the increased intensity of smoothing, as the proportions of true maxima were over 90% on these plots, which is also a mark of intensive smoothing.

The original idea was to use automatic methods to classify deciduous and coniferous trees from aerial images, the classification eventually had to be done visually due to problems in combining the data sets. The problem was radial displacement which causes tree tops in aerial images not to align properly with ALS data. The weaknesses of the method used were that it is not objective and that the manual work involved is time consuming. Also, the radial displacement caused some problems in visual classification, as it was not always clear which was the canopy that was to be classified in the aerial image, even if there were several images that could be used. Canopy segments obtained from the CHM and tree canopies shown in aerial images did not match completely, or at all in some cases, and brightness values differed between the images and within an image. This meant that there was variation in radiometric values and texture even among the canopies of the same species, which complicated visual interpretation. Also, some of the trees were completely shadowed by larger trees. As a result, 74% of the tree canopy segments were correctly classified as coniferous or deciduous trees.

Three-way tree species classification (pine, spruce and deciduous trees) using aerial images has been reported by Haara and Haarala [2], Persson *et al.* [19] and Korpela *et al.* [8], for example. When comparing the present results with these, it should be noted that the classes, methods and airborne data are of a different kind. The results of Haara and Haarala [2] were slightly better than ours, in that 79-90% of the trees were classified correctly when training data from the same image were used and 59-83% when the training sets originated from the other images. Persson *et al.* [19] achieved an accuracy level of 90%, and although two of the classes (pine and spruce) have relatively similar radiometric values, misclassifications between them were still only 5% (pine classified as spruce) and 14% (spruce classified as pine), while 6% of the deciduous trees were misclassified. Korpela *et al.* [8] achieved an accuracy level of 93.7% by visual tree species classification in managed forests.

The separation of aspens from other deciduous trees entailed the use of a total of four predictor variables in a discriminant function and achieved a classification accuracy of 78.6%. These predictors describe the characteristic shape of the tree crown and differences in reflected pulse intensity (f_i90) between the species in question. Higher proportions of vegetation hits were obtained for aspens than for the other deciduous trees, which means that the others are characterised by a more star-like and less uniform crown. The proportions of laser points reflected at the 60^th^ height percentile indicate smaller crown heights in aspens, since lower values were obtained for this species on average. This was also discovered in the field measurements. The standard deviation of the heights of the reflected first pulses was also greater for aspen, which may indicate that the crowns more often have several tops or that some of the reflections originating from the tops of large side branches. Finally, the intensity at the 90^th^ height percentile was lower for aspen than for the other deciduous trees.

The aspen trees in this area were on average larger than the other deciduous trees and the aspens were classified more precisely, although the average crown area of the misclassified trees was larger in the case of the first function. This may have been caused by the intensive smoothing, causing the number of local maxima to be relatively small and some of the segments to be too large as a result (see Figure 3). There are likely to be more large segments formed from the canopies of two or more trees among the misclassified trees than among the correctly classified ones, and it is probable that the misclassifications can partly be explained by the variation in dimensions between individual trees, especially among those classified as 'other deciduous trees'. As shown in Figures 1 and 2, the distributions of misclassified trees are narrower than those of the correctly classified trees, and the successful classification of the smallest trees in particular is somewhat surprising. The numbers of trees in the smallest and largest height and DBH classes are not high, however.

Holmgren and Persson [4], who classified pines and spruces using linear and quadratic discriminant analyses with varying numbers of predictors, obtained their best results (94.8% correctly classified) with six predictors (standard deviation of the intensity of the returned pulses, proportion of first returns, proportion of surface hits, mean value of the parameters of the parabolic surface, relative standard deviation of laser heights, and the 90^th^ height percentile divided by the estimated tree height). The absolute value for the correlation between the predictors was at most 0.70. The better results of Holmgren and Persson [4] relative to ours may be partly explained by the differences in crown shapes between the species concerned. As Holmgren and Persson [4] pointed out, spruce is usually more conical than pine, especially in the case of older trees. With aspen, birch and alder it is more difficult to implement such a generalization regarding crown shapes. Brandtberg [14] classified three deciduous tree species using ALS data with a point density of 12/m^2^ and achieved an accuracy of 64%, which is a little less than here. It should be noticed though, that he had three classes whereas we needed only two. Moffiet *et al.* [13] demonstrated that the proportion of singular laser hits had the strongest discriminating power in the classification of poplar box and cypress pine-dominated forest sites. Stand level classification clearly differs from tree level classification, however. Nevertheless, similarities can be found in the predictors, as the proportion of singular returns had most of the discriminatory power in analysis of Moffiet *et al.* [13], whereas the proportion of vegetation hits was an important predictor for us.

Radiometric values and textural features [36] of digital images used with ALS data variables could improve the results. The results of the tree species classifications by Haara and Haarala [2] and Persson *et al.* [19] show that those based on aerial images can achieve high accuracy levels with the three most common Scandinavian tree species. A combination of ALS data variables and aerial image variables could be very efficient, since both the shape properties and radiometric characteristics of the crown would then be utilized.

The intensity values and the standard deviation of the intensity vary from one tree species to another and between forest sites with different trees dominant [4, 13, 17]. In addition, intensity-based variables had discriminating power in the case of the preliminary discriminant functions used here. There are many factors affecting intensity values, however, and more research into intensity-based variables may be of help in classifying trees to species.

Future developments in the airborne laser scanning technique will undoubtedly ensure not only that denser data can be obtained at reasonable cost, but also that the crown structures of individual trees can be described more accurately. In addition, the development of full waveform lidar could provide suitable solutions for tree species classification in the future [37].

## Figures and Tables

**Table 1.** Main characteristics of the forest stands.

**Variable**	**Tree species**	**Mean**	**Min**	**Max**	**Std**
Volume, m^3^/ha	Total	333.3	79.0	412.9	82.2
	Aspen	123.7	42.6	287.6	17.9
Number of stems/ ha	Total	1167.9	433.3	2883.3	562.1
	Aspen	269.4	11.1	1316.7	329.5
Mean height, m	Total	14.5	8.9	16.4	2.3
	Aspen	22.4	16.1	28.0	2.9

**Table 2.** Parameters used in height based filtering: the smoothing intensity in the lowest height class (low) and in the highest class (high), and the number of height classes (height fixed to 6 meters).

**Sample plot**	**σ low**	**σ high**	**Number of height classes**
1	0.4	2.2	4
2	0.8	1.8	5
3	0.4	1.6	4
4	1	1.1	5
5	0.8	1.3	5
6	1	1.1	5
7	0.4	2.4	5
8	0.4	1.8	5
9	0.4	1.2	5
10	1	1.2	5
11	0.4	1.3	5
12	0.6	1.8	4
13	0.4	1.4	4
14	0.4	1.6	4

**Table 3.** Results of the classification into deciduous and coniferous trees.

**Sample plot**	**Canopies**	**Correctly classified**	**%**
1	17	13	76%
2	22	18	82%
3	28	22	79%
4	27	19	70%
5	34	28	82%
6	33	18	55%
7	25	24	96%
8	22	16	73%
9	28	21	75%
10	48	36	75%
11	52	41	79%
12	45	29	64%
13	21	13	62%
14	16	10	63%

Total	418	308	74%

**Table 4.** Proportions of correctly classified trees on each sample plot

**Sample plot**	**Trees to be classified**	**Correctly classified**
1	1	100%
2	3	100%
3	15	60%
4	7	71%
5	15	60%
6	10	70%
7	14	79%
8	12	83%
9	14	86%
10	33	79%
11	32	91%
12	22	73%
13	11	91%
14	7	86%
